# Testosterone Responses to Intensive, Prolonged Endurance Exercise in Women

**DOI:** 10.3390/endocrines1020011

**Published:** 2020-11-05

**Authors:** Anthony C. Hackney, Hannah N. Willett

**Affiliations:** 1 Department of Exercise & Sport Science, University of North Carolina, Chapel Hill, NC 27599, USA;; 2 Department of Nutrition—School of Public Health, University of North Carolina, Chapel Hill, NC 27599, USA

**Keywords:** hormones, anabolic, physical activity, endocrine, female

## Abstract

**Objective:**

To examine the response of testosterone in women to an intensive, prolonged endurance exercise bout that mimicked a competitive event.

**Methods:**

Ten healthy eumenorrheic women ran to exhaustion at ~100% of their ventilatory threshold in their follicular menstrual cycle phase. Testosterone measures were assessed pre-exercise, immediately, 30 min, 60 min, 90 min, and 24 h post-exercise.

**Results:**

At exhaustion (75.1 ± 7.0 min), total (56%), free (36%), and bioavailable testosterone (50%) were increased from pre-exercise values (*p*< 0.05). At 24 h post-exercise, these measures were decreased from pre-exercise values (−21%, −31%, −18%, respectively; *p* < 0.05). Effect sizes for these changes ranged from medium to large in magnitude.

**Conclusion:**

Testosterone was elevated in the early recovery period following exhaustive endurance exercise but was reduced by 24 h afterward. These outcomes are comparable to responses seen in men when sex-based concentration differences are considered.

## 1. Introduction

Anabolic hormones exert a biological effect on many tissues in the human body [1]. For example, they are important for the growth and maintenance of skeletal muscle, enzymatic proteins, bone, and red blood cells as well as helping to enhance neural function [1,2]. Testosterone is a key steroid-based anabolic hormone that induces many of these biological effects in both men and women [1]. It is also a hormone that plays a role in the adaptive process to exercise training [1,2]. The exercise role and response of testosterone is well known in men but to a much lesser degree in women [3]. Because testosterone is associated with the muscle hypertrophy response to exercise, many exercise studies have examined its response to resistance, strength-based activities. Research on the effects of prolonged endurance exercise on hormones, such as testosterone, are less frequently studied in both men and women, even though the evidence clearly points to the necessity of testosterone in the physiological adaptations of endurance-based activities [4,5].

The worldwide involvement of women in endurance sporting activities, such as competitive marathons and triathlons, has grown exponentially over the last few decades [6]. This expanded involvement has led to calls for more research on women athletes [7]. This seems especially warranted in competitive sporting-like scenarios that place great demands and stress on athletes [6,8]. This last point is an important consideration, since intensive competitive exercise provokes differing endocrine responses than non-competitive exercise situations [8]. Therefore, the purpose of this study was to examine and describe the response of testosterone in women to an intensive, prolonged endurance exercise bout to exhaustion which mimicked a competitive event.

## 2. Materials and Methods

### 2.1. Study Participants

Young, healthy eumenorrheic women ages 18 to 26 and not using oral contraceptives participated in this study (*n* = 10; see Table 1). The subjects were aerobically trained endurance runners or triathletes, having trained for a minimum of 2 years, 5 days per week for at least 30 min a day. Participants were asked to complete 3 sessions in our laboratory for the study. These sessions all occurred in the early follicular phase of the menstrual cycle. Cycle phase lengths were confirmed by oral temperature records for ~2–3 months before the study.

### 2.2. Study Protocol

#### 2.2.1. Session One

Subjects completed a written informed consent document, a training history questionnaire, and a medical history questionnaire. Height (cm) and body mass (kg) were recorded. Skinfolds were assessed at three sites (i.e., triceps, supra-iliac, and femoral; Harpenden calipers, Creative Health, Ann Arbor, MI, USA), and the Jackson-Pollock-Ward regression equation was used to estimate body composition [9]. A modified Åstrand treadmill test was used to determine each subject’s maximal oxygen uptake (VO_2max_) with continuous respiratory gas measurements using a TrueMax 2400 analyzer system (Parvo Medics, Sandy, UT) [10]. Heart rate (HR; bpm) was recorded every minute of exercise, and a rating of perceived exertion (RPE) was recorded at the end of every three minutes. The grade of the treadmill was kept constant at 1.5%, while the speed was increased incrementally every 3 min. The VO_2max_ test was terminated upon volitional fatigue of the subject and published criteria used to valid maximal effort [10]. This session occurred between days 1 and 2 after the end of menses blood flow and in the afternoon between 12:00 and 15:00 h.

#### 2.2.2. Session Two

Before the second session, subjects were asked not to participate in any strenuous activity for 24 h before, ingest any alcohol, or to ingest any caffeine for the 12 h before. For three days before the second session, the subjects were asked to eat a diet with ≥60% of their caloric intake from carbohydrates. An interview-based 3 day dietary recall at the beginning of the second session confirmed dietary guidelines were met.

Session two took place 3 to 4 days after session one and occurred between 12:00 and 15:00 h with the subjects fasted. A catheter was inserted in an antecubital vein for blood sampling. Subjects then rested supine for 30 min, and a baseline pre-exercise blood sample (3 mL) was taken. Subjects were fitted with an HR monitor (Polar, Finland) and began a warm-up session, which consisted of cycling on an ergometer (Monark 814 Ergomedic, Sweden) for ~5 min followed by ~5 min of stretching. They then began their run on the treadmill at a speed to elicit their pre-determined ventilatory threshold (±5%), maintained until they reached exhaustion. The ventilatory threshold (VT) for each subject was determined from their VO_2max_ test [10]. Respiratory gases, HR, and RPE data were collected at 5, 30, and 60 min during the exercise to monitor intensity. After 60 min, these measures were taken every 15 min and again at exhaustion (for this brief report, only select responses are reported).

At exhaustion, a post-exercise blood sample (3 mL) was taken immediately. The subjects then rested supine for 90 min. Additional blood samples (3 mL) were taken at 30, 60, and 90 min during this post-exercise recovery period. Throughout the exercise, subjects were verbally encouraged, and as they approached the point of exhaustion, this encouragement was increased to simulate what might occur during a sporting competition. Throughout the exercise and recovery period, the subjects were allowed to drink water ad libitum.

#### 2.2.3. Session Three

Session three took place 24 h after the exhaustive run termination in session two. The subjects were instructed not to ingest any alcohol or caffeine or participate in any strenuous activity between sessions. Once at the laboratory, the subjects rested supine for 30 min, and then a final blood sample (3 mL) was taken.

### 2.3. Biochemical Analysis

Blood samples were collected in sterile tubes (BD Systems, Franklin Lakes, NJ, USA) and placed on ice immediately. A small sample of whole blood was transferred into EDTA-treated tubes (BD Systems) to be analyzed for hematocrit and hemoglobin, from which plasma volume shifts were calculated using the Dill and Costill [11] equation to account for hemoconcentration of the blood. The remaining whole blood specimens were centrifuged at 3000× *g* at 4 °C to separate sera. Testosterone, sex hormone-binding globulin (SHBG), and albumin were assessed with commercial analytical kits (DPC Inc., Los Angeles, CA, USA; Abcam, Cambridge, MA, USA). Additionally, free testosterone and bioavailable testosterone were calculated using procedures from Vermeulen et al. [12].

### 2.4. Statistical Analysis

This pilot study used a repeated-measures research design with pre-exercise responses serving as the comparison (i.e., reference point) for all sequence responses over time in each dependent variable (i.e., hormonal-related measures). Statistical analyses were conducted using the SPSS software program (version 25, SPSS Inc., Chicago, IL, USA). Descriptive statistics were determined for all measurements. Hormone values (corrected for plasma volume [PV] shifts, which are what are reported herein) were not normally distributed; thus, we utilized a Friedman ANOVA with pairwise signed-ranks tests for post-hoc procedure analyses. Statistical significance was set at *p* ≤ 0.05, and Hedge’s *g* was used to calculate effect size (ES) [13].

## 3. Results

Exhaustion occurred at a mean time of 75.1 ± 7.0 min (±SE) at a mean running speed of 12.0 ± 0.2 km/h. At the end of exercise HR = 177.3 ± 3.5 bpm, RPE = 15.8 ± 0.6 Borg units, VO_2_ = 2.36 ± 0.08 L/min which represented 98.8 ± 2.4 % of their VT.

Table 2 presents the hormonal and binding protein changes before and in response to the exhaustive exercise into recovery. At the end of the exercise, total, free, and bioavailable testosterone were significantly increased from resting, pre-exercise values (*p* < 0.05). Sex hormone-binding globulin and albumin displayed similar responses (*p* < 0.05).

After these significant increases immediately post-exercise, all measurements gradually decreased, returning to pre-exercise levels by 90 min post-exhaustion. At the 24 h post-exercise time, total, free, and bioavailable testosterone reached significantly lower levels than their resting, pre-exercise values (*p* < 0.05). In contrast, SHBG and albumin were elevated at 24 h post-exercise (*p* < 0.10 > 0.05), albeit these differences were not statistically significant.

The ES for all these changes are also reported in Table 2. This analysis demonstrates that for the significant changes in our measures, the ES ranged from medium to large in magnitude.

## 4. Discussion

Our purpose was to examine and describe the response of testosterone to intensive, prolonged endurance exercise in women that mimicked a competitive sporting event. Women have traditionally been an understudied population in exercise research [6]. This occurrence is despite the exponential growth in the involvement of girls and women in physical activity and sports over the last several decades [7]. The testosterone response of men to nearly all forms of exercise is well defined, but not so in women, even though testosterone has physiological effects in women just as in men [1]. Thus, our research group wondered if the behavior of testosterone was similar in women as that in men when performing strenuous exercise, specifically endurance activities. The latter was of interest as testosterone responses to endurance activities are studied less often compared to resistance-based exercise.

The present hormonal findings for testosterone are remarkably comparable in nature relative to similar male-based research. The published findings of Anderson et al. [14], Keizer et al. [15], and Hloogeveen et al. [16] all show alike steroid hormonal changes in men as we observed in our female subjects. That is, prolonged endurance exercise produces a biphasic response in testosterone in which, initially, there are increases from the exercise followed by reductions in recovery. Although, it is important to recognize that the magnitude of some of the absolute hormonal concentration changes observed in these studies vary from those of the present data due to the fact of sex-related differences [1]. Furthermore, the ES magnitude of our hormonal responses (i.e., of the significant testosterone responses) ranged from medium to very large for the observed responses in our subjects [13]. Since ES is independent of sample size, the magnitudes of the ES values suggest our findings are more robust than what the significance testing (*p* levels) implies [13].

Perhaps the most interesting finding within our results was the tendency for testosterone to be reduced 24 h into the recovery from the exercise. It is unclear if the observed reduction is a feedback regulatory suppression following the acute substantial hormonal increases in response to the exercise [17], the consequence of a delayed increase in the metabolic clearance of the hormone, or perhaps a stress reactivity response (e.g., due to the fact of a cortisol-induced inhibition) and, as such, a dysfunctional response [4,17]. Conversely, Kraemer et al. [18] has proposed these type changes are reflective of the homeostatic mechanisms involved in the repair and recovery process. Our finding of this biphasic hormonal responses, leading to the reduction in testosterone during recovery, needs to be addressed in future research in women so the underlying rationale and mechanism can be identified.

We recognize our study was limited due to the presence of several factors: (1) its small sample size, (2) its relatively narrow range of biomarkers assessed, and (3) the fact that we only examined women in the follicular phase of their menstrual cycle. Further research is recommended to expand upon our work and overcome these limitations.

In conclusion, we found in exercise-trained women, testosterone remained elevated in the early recovery period following exhaustive endurance exercise but was reduced by 24 h afterward. These outcomes are comparable to similar types of exercise responses seen in men when sex-related concentration differences are taken into account.

## Figures and Tables

**Table 1.** Characteristics of the study participants.

Measurement (Units)	Mean	Standard Deviation
Age (y)	23.5	2.1
Height (cm)	165.7	6.2
Mass (kg)	59.8	3.8
Body Fat (%)	19.4	3.7
Training (y)	6.5	3.1
VO_2max_ (L/min)	3.18	0.22
VO_2max_ (ml/kg/min)	56.2	2.7
VO_2_ at VT (L/min)	2.39	0.27
VO_2_ at VT (% of VO_2max_)	76.2	6.2

VT = ventilatory threshold.

**Table 2.** Testosterone (T) concentrations after the prolonged run to exhaustion (mean ± SE).

Hormone	Pre-Exercise	Exhaustion (Post-Exercise)	30 min, Post	60 min, Post	90 min, Post	24 h, Post
Total T (ng/dL) ES	17.7 ± 2.2	27.6 * ± 3.2	24.7 ± 2.0	22.4 ± 2.9	19.5 ± 2.2	13.9 * ± 2.0
		1.04	0.96	0.53	0.23	0.52
Free T (ng/dL) ES	0.212 ± 0.023	0.289 * ± 0.017	0.273 ± 0.034	0.252 ± 0.020	0.220 ± 0.033	0.146 * ± 0.031
		1.10	0.60	0.54	0.08	0.70
Bioavailable T (ng/dL) ES	4.62 ± 0.31	6.92 * ± 0.51	6.27 ± 0.61	5.98 ± 0.59	4.90 ± 0.39	3.81 * ± 0.29
		1.58	0.99	0.84	0.23	0.78
SHBG (nmol/L) ES	62.1 ± 5.1	72.1 * ± 3.1	68.1 ± 6.2	67.2 ± 4.9	66.9 ± 5.0	69.8 ± 2.9
		0.69	0.30	0.30	0.28	0.54
Albumin (g/dL) ES	4.0 ± 0.3	4.7 * ± 0.2	4.2 ± 0.5	4.4 ± 0.4	4.1 ± 0.70	4.7 ± 0.3
		0.79	0.14	0.32	0.05	0.67

* significant difference from pre-exercise, resting values, *p* ≤ 0.05; ES = effect size, sex hormone-binding globulin = SHBG, Hedge’s *g*
